# A Treatise on the Diseases of Infants, Founded on Recent Clinical Observations and Investigations in Pathological Anatomy; with a Dissertation on the Viability of the Child

**Published:** 1840-04-01

**Authors:** 


### A Treatise on the Diseases of Infants, founded on recent Clinical Observations and Investigations in Pathological Anatomy; with a Dissertation on the Viability of the Child. By *C. M. Billard*, D.M.P. Translated from the Third French Edition, with an Appendix, by *Jas. Stewart*, M.D. (New York.) Churchill, London, 1839. Pp. 620.

This American translation of Dr. Billard's elaborate work, will supply a want felt to exist in our medical literature. It is true, we possess a mass of valuable practical information—the result of accumulated and careful clinical observation—which enables us to treat the diseases of children with a degree of success unknown formerly, and unsurpassed by the practitioners of other countries. But, owing to our very limited means of investigating the nature of these affections after death, many of our opinions rest upon very unsatisfactory and unscientific bases. Dr. Billard has enjoyed opportunities of pursuing pathological investigations to an almost unlimited extent, at the Hospice des Enfans Trouvés of Paris; and he has very diligently availed himself of them,—presenting to the world, as the result, a book remarkable for the variety and importance of the facts it contains. His attention has not been confined solely to the diseases of young infants; but he has succeeded, by means of his numerous observations upon the normal and abnormal state of their various organs, in establishing, upon a firm basis, many points in physiology and medical jurisprudence, concerning which the most vague opinions had heretofore prevailed.

When we mention also, that the book contains nearly 100 cases of diseases occurring in very young infants; (our annals of similar cases, carefully recorded, being very scanty,) and, that it may be considered, almost as a complete manual of information, respecting congenital disease and malformation, our readers will allow that it is no common work; and that it justifies our devoting a considerable space to its consideration.

But, while we rejoice that Dr. Billard has thus diligently availed himself of the opportunities presented to him, do we regret, that such do not exist in our own country? Indeed, no! Much as we may, in other respects, be disposed to envy our continental brethren, their superior means of carrying on anatomical and pathological enquiries, we do not do so in the present case. The French system of Foundling Hospitals, not only produces a lax morality, by offering every facility to the reckless incurring, and heartless abandonment of maternal responsibility—to the severing of those ties between mother and child, which the very brute creation teaches us to respect; but also, by the congregation of young children, which it renders unavoidable, becomes itself a frightful source for the generation of infantile disease and mortality.*

* In the progress of his work, Dr. Billard frequently explains the severity and mortality of various diseases by the vitiation of the air, consequent on these large assemblages of children, and the deficient and improper nourishment to which they are more or less subjected. M. Benoiston de Chateauneuf states

We will now proceed to the analysis of the work; presenting to the reader all points which are novel, or likely to be interesting.

### PART I. OF THE PHENOMENA WHICH ARE PRESENTED UPON EXAMINING EXTERNALLY THE CONDITION OF THE CHILD.

### 1. *Attitudes of the Child.*

This is but a meagre chapter, and we only notice it for the sake of an useful caution, as to holding infants in the erect posture too soon; a practice too frequently followed.

"At six weeks or two months, the infant scarcely begins to have power to support its head, but is constantly to be seen moving it about in a very irregular manner; it appears too heavy for the muscles of the neck to support its movement.

We are of opinion, that the infant ought to be kept in the horizontal position, or carried on a pillow, when it is perceived that it has not as yet sufficient muscular strength to maintain the head in an erect position: in about two months, it will begin to have power sufficient for that purpose. To this rule, however, there are many exceptions, attributable to the strength or weakness of different individuals. The vertebral column becoming more and more solid, the trunk sustains itself better, and at the age of four or five months, we see the infant support itself in sitting." 11.

### 2. *Colour of the Skin.*

"When the finger is applied to the skin of an infant, the red colour disappears at this point, and it becomes yellowish; afterwards the blood returns by degrees in the capillaries from which the pressure had removed it, and the yellow tint is replaced by the previous red. It will often be observed, after the red colour has disappeared, and before it becomes altogether white, that the skin will exhibit a universal tint of yellow, and sometimes of a copper colour. This is thought, by physicians generally, to indicate an affection of the liver. The result of our observations on this subject, however, will justify us in controverting this opinion."

"About the end of the third month, the distinctive colour becomes established, and we can, from that time, distinguish dark-complexioned children from those that are fair; at a much more tender age the hair has already assumed its proper hue; but it is not until the period of which we are speaking, that the skin on the body becomes dark or light coloured, the face either pale or ruddy, and the traits peculiar to each constitution delineated." 13, 14.

### 3. *Separation of the Umbilical Cord.*

(*a.*) *The Desiccation.*—This process commences at the distal end of the

---

(Annales d'Hygiène, tom. 21,) that, between the years 1824 and 1833 were received into the French Foundling Hospitals 336,000 infants, making, when added to those already there in 1824, (116,000,) in all 452,000. During these ten years, there died in the hospitals 46,755, and at nurse 151,750; in all 198,505. He states also, the number received in 1837 to be 127,000, and calculates their expenses at eleven million francs.

Here is indeed a wide field for pathological research!

cord, usually on the first or second day, and is completed on the third, although these periods are sometimes later. It consists in a drying up of the gelatine of Wharton, which then compresses the vessels; these become flat and tortuous, and are seen as small, black filaments, winding in the midst of a semi-transparent substance.

This desiccation, apparently a mechanical, is actually a vital process, since, in the still-born child, instead of the cord drying and separating in a few days, it undergoes an entirely different process, viz. a perfect decomposition. "When the cord is left to putrefy upon the dead body, it first assumes a greenish white colour; then becomes corrugated at its extremity, and shrinks. Its pellicle is easily detached, but the cord will not separate from the abdomen at its point of insertion, as is observed to be the case during life. I have never seen the cord of a still-born child become dry before the fifth or sixth day, and have also observed, that it preserves its circular form and suppleness for some days." From this fact arises the importance of noting the desiccation of the cord in reference to legal medicine. His conclusions are:—" 1. The desiccation of the cord cannot take place except during life. 2. At the period of death the desiccation is suspended, or considerably retarded. 3. If the cord be fresh, or the shrinking but just commenced, the infant may have been still-born, or have lived but a very short time. 4. If the cord has already exhibited the beginning of desiccation, or is completely dry, the infant has lived at least one day; the more recent the death of the fœtus, the greater is the dependence upon these conclusions." Dr. Stewart remarks judiciously upon the above observations, that, though in the majority of cases they are applicable, yet, that in medico-legal investigations, they should be considered as only corroborative testimony.

(b.) *The Separation.*—Although, usually occurring at the fourth or fifth day, there are so many exceptions, that we cannot draw any useful conclusion upon the subject. The separation is not effected by the pressure of the skin, but by that produced by the indurated gelatine; and, in proportion as the cutaneous ring surrounds the insertion of the cord closely, there will be a degree of inflammatory action present after the separation, though Dr. Billard by no means agrees with many authors, who believe such *inflammation* to be the cause of the separation; he considering it as merely accidental. In 86 infants, he found inflammation present in 25, and absent in 61. Indeed, when inflammation and suppuration were present, the process of separation was always retarded; for the funes have then, generally, a broad attachment, and contain a larger quantity of gelatine; the desiccation of which, is a necessary preliminary to the separation. "We should not, therefore, attach much importance, as has been done by some distinguished writers on legal medicine, to the absence or presence of a red circle around the umbilicus, when we are examining the dead body of an infant to determine whether it died before, during, or after birth."

Dr. Stewart says, in the Appendix:—"We can scarcely coincide with the opinion, that the separation of the cord arises from mechanical causes: the appearances of inflammation are of still too frequent occurrence to attribute it to these causes."

(*c.*) *Cicatrization of the Umbilicus.*—Generally, when the ring is thin, and the cord slender, the cicatrization will occur about the ninth day; but when we meet with a very projecting umbilicus, as it corresponded probably with a thick cord, we may conclude the cicatrization was not completed until after that period.

(*d.*) *Hæmorrhage from the Cord.*—Dr. Stewart relates a case of fatal hæmorrhage, arising from an œdematous state of the cord, the ligature on which became loosened by the oozing of fluid. We may here notice a state of the funis we lately met with, viz. an inordinate thickness of that part, arising, not from œdema, but from the presence of a large quantity of gelatinous matter, of an infinitely less dense texture than ordinary; so that, even a tape ligature, when applied with the slightest pressure, cut through it like a knife. Had hæmorrhage existed, it could not have been restrained by any ligature; but, on examining the cut surface, the orifices of the vessels were scarcely visible.

4. *Exfoliation of the Epidermis.*

This, which differs in toto from the separation of the epidermis which results from putrefaction, he has never known occur prior to birth, or in premature children. The period of its occurrence, after birth, as observed in eighty-six children, was uncertain, although, from the third to the fifth day, was the time when it was generally at its height. Its duration, also, is very various, continuing in some cases for a month or two; and it is both of longer duration, and of a more marked character, where marasmus is present, owing to the dryness and flaccidity of skin this produces.

" There exists in *adults* an analogous phenomenon, where disease has produced a rapid emaciation. In the course of their convalescence, to use a popular phrase, they change their skin. It appears as if nutrition had been entirely suspended in those parts in which it naturally proceeded in a feeble manner, and that the epidermis being thus deprived of its portion of blood, had become withered and dead. The *cause* of the epidermic exfoliation in new-born children, is susceptible of a satisfactory explanation. The integuments of the infant are for about seven months immersed in a liquid which keeps them moist and supple. The epidermis, until the period of birth, is, as it were, soaking in the waters of the amnios. When exposed to the air, it becomes suddenly dried, and loses the suppleness peculiar to it during the intra-uterine existence. The epidermis is very soon *reproduced* in those parts which are most exposed to the air, but the folds of the skin, as in the arm-pits, the neck, or groin, or any other parts deprived of a direct contact with the air, secrete a fluid like that formed by mucous membranes. This is easily dried up, in assisting the formation of a new epidermis by the use of absorbing powders. This circumstance serves to prove that the epidermis is only a cutaneous secretion; a covering almost inorganic, being destroyed and re-established according as the cutaneous surfaces are exposed to the action of the air, or are kept from its direct influence." 34.

5. *The Cry of the Infant.*

Dr. Billard analyzes the cry into two parts; the cry *proper*, which is heard during expiration, sonorous and prolonged; and the *reprise*, more acute, less perceptible, and occurring during inspiration; " it is an interval, a sort of effort at renewal between the cry just finished, and that about being commenced." It is from modifications of these two parts, he draws his signs. When the reprise is silent, while the cry is distinct, the lungs are healthy, and quite permeable to air: the child does not then employ all its respiratory efforts, so that, the air traverses the glottis without noise, which is only produced when it passes in expiration. On the other hand, in cases where the cry is smothered, and the reprise alone heard distinctly, there is almost always pulmonary inflammation or engorgement. In twenty children examined, who possessed this modification of the cry, impediments to a free ingress of air were found in the lungs. He thinks, the reprise should be well observed, by those called upon to give an opinion upon the viability of a child. In *tone*, he says, the reprise becomes *acute*, in those affections, in which the renewal of respiration is especially urgent, as, affections of the tonsils, larynx, and trachea;—hence, the crowing in croup, in which the cry proper is replaced by a blowing sound. The *husky* tone attaches most to the cry proper, and is heard in affections of the bronchiæ and larynx. In three cases, in which the cry was faint, trembling, or jerking, like a *goat's*, he found œdema of the glottis. The *duration* of the two parts of the cry is various. When they follow each other rapidly, or when three or four cries precede one reprise, sudden and violent pain is usually denoted, as from a pin, burning the mouth, colic, &c. The interrupted cry exists in angina suffocativa. In conclusion, Dr. B. says—" The changes in the proper cry usually indicate an affection of the lungs and bronchiæ, whilst the changes in the reprise are ordinarily evidences of an affection of the trachea and larynx."

### 6. *Absence of Tears in Infants.*

" It is an important fact to notice here, that very young children never shed tears while they cry, or at least, it is of very rare occurrence. The secretion of the lachrymal gland is excited, as is well known, immediately and sympathetically by sorrow; but are children at the tender age of which we are speaking, under the influence of the mental emotions? Is this secretion produced by any other influence than the nervous excitability proceeding from some moral cause? And are physical distresses, which appear to be the only kind endured by a being whose brain cannot as yet combine ideas, and from which there appears to emanate no volition, capable of acting on this gland? These are questions which it is difficult to answer. The lachrymal gland at this period is perfectly developed, it receives arteries and nerves, and does not differ anatomically from other glands. Notwithstanding this, no tears flow while the child is crying; and while the simple recollection of a lost friend, dear to us, will fill the eyes with abundance of tears, yet, in the young infant, in spite of the reiterated cries from sleeplessness, sickness and pain, the lachrymal secretion remains undisturbed. This fact is a remarkable example of the particular influence of the nervous system on the functions of certain organs." 41.

### 7. *State of the Pulse in Children.*

In England, practitioners, in general, are accustomed to attach very little importance to the pulse, as a guide for the treatment of the diseases of very young children; it may be, that they attach too little; but certainly we are at a loss to understand, how Dr. Billard is enabled (he however acknowledges the difficulty) to distinguish the jerking, the thread-like, or the easily compressed pulse. His fingers must have acquired a nicety of touch denied to ordinary men; but, in order to assist those who may wish to attain a little tact, we transcribe his directions for feeling the pulse of young infants.

"The physician ought, if possible, to avoid grasping and fixing the arm of the child with his hand, as continual motion will then be made to relieve it from the compression of the fingers: it is much better to permit the arm to remain free, and to apply the fore-finger in the track of the radial artery: it will not be discovered until after a little search: at the same time care should be taken that it be not strongly compressed, as it might thereby become flattened and its pulsation thereby rendered imperceptible. After having felt a few beats, the pressure of the finger ought to be gradually diminished until the artery is permitted to dilate to its full volume. I have remarked that it is much easier to find the artery by applying the index finger alone, than by placing the first three fingers on the track of the artery, as is recommended in adults. The finger can in the same manner be applied to the temporal artery." 55.

The common idea of the *frequency* of the young infant's pulse, his observations prove to be incorrect; for among forty children, between one and ten days old, he found, in about one half, the pulse was nearly as slow as in the adult; of thirty-five between one and two months, fifteen were in a similar predicament; but, of eighteen children between two and three months, all but three exceeded the adult pulse; which was also the case in the few he examined above one year. Hence, "it appears that the pulse of a very young infant is often not much more frequent than that of an adult, but that it increases in frequency in proportion as the child advances in age; whence it follows, that it is wrong to assert, in a manner so exclusive and general as is usually done, that the pulse in children is more frequent than in adults."*

### PART II.—DISEASES OF INFANTS.

The extensive scope of the author's undertaking may be judged of by the following passage.

"If it is our wish to investigate the affections of the different organs of children, it will be necessary to point out the principal congenital malformations, together with the alterations of texture which each organ undergoes during intra-uterine life. It is this which I now propose to do; and also to describe the symptoms by which, at the birth, a correct diagnosis of the various alterations may be made; and considering in their course those diseases which are developed after birth. The therapeutic agents necessary for their relief will conclude the history of each disease." 61.

* The observations of Dr. Naegele, in his work on Obstetric Auscultation, will still farther modify the general opinion upon this subject. After noticing the irregularities of the contractions of the fœtal heart, he continues "how strangely does the pulse of children vary during the first few years of their life! To-day it beats 80 in the minute; to-morrow, although there may be no perceptible difference in the state of the child, the pulse has risen to 160 or 180; it varies with extreme rapidity during tranquil sleep, as well as when the infant is awake. If the child move, or take food, or if it be under the influence of any mental impression, the pulse rises 30, 40, or 60 strokes in the minute; and this variableness while it would confound the inexperienced has taught those who are well acquainted with children's diseases, not to draw their conclusions from the state of the pulse."—*West's Translation*, 33.

1. *Diseases of the Skin.*

As the descriptions are almost all founded upon those of Willan and Rayer, and the treatment not different from that usually adopted, we shall not notice many of the affections treated of in this chapter. He classifies these diseases in an ingenious manner, in imitation of Lamarque's Botanical System; so that their various affinities and contrasts are displayed in parallel columns.

1. *Petechiæ.*—There is an interesting case of this affection, in a child three days old. The whole skin was unequally spotted, like a tiger's. The child died in two days. The alimentary canal was covered with petechial spots, and it contained a considerable quantity of effused blood; the spleen was large, gorged and ruptured; the heart large and congested, and its surface covered with petechiæ—bladder and kidneys ecchymosed—brain much congested—effusion of blood into the cellular tissue of trunk and limbs. In another case, of a child aged eight days, the petechiæ gradually faded away, and the child recovered. The author recommends, when the petechiæ are accompanied by symptoms of congestion, that a few leeches be applied to the arms; and that where this is not the case, we should leave the case to Nature.

2. *Congenital Eruptions.*—The author's observations are chiefly confined to congenital *small-pox;* he relates the history of the fœtus, covered with small-pox pustules, preserved in Sir Astley Cooper's Museum. Both the author and Dr. Stewart supply references to other cases, some of which we subjoin.*

3. *Syphilitic Eruptions.*—Dr. Billard justly observes, that it is too much the practice to set down various eruptions of young infants indiscriminately as syphilitic. He is perfectly right in saying, that various eruptions both congenital and occurring after birth, are thus erroneously termed: but yet many of them are really syphilitic; and we are much surprised, that this work (composed almost, as it were, in the wards of a hospital, the great bulk of whose inmates must be derived from the lowest classes of society,) contains little or rather no information upon the subject of syphilis: whether, as it manifests itself at, or shortly after, the birth of the infant infected in utero; or later, when the affection has been communicated by a diseased nurse.†

4. *Erysipelas in Infants.*—In the year 1826, thirty cases fell under the author's care; and of these 16 died, in all of whom marks of enteritis were found. In the treatment of the affection, Dr. B. seems to us to place too

---

* Dyckman's Pathology of the Human Fluids, New York, 1814; Francis' American Med. and Phil. Register, Vol. 4; Duncan's Medical Commentaries, Vol. 19, which contains many additional references; Memoirs Lond. Med. Society, Vol. 4; Phil. Trans. abridged, Vol. 3, p. 308; Boerhaave's Van Swieten.

† See Lawrence's Lectures in Lancet, Nos. 340 and 400.

little reliance upon aperients, and other general means, and too much upon local treatment. In the severe cases he recommends scarifications, and sometimes blisters; both of which means, it would seem to us to be rarely applicable to young infants. Dr. Stewart observes, that this disease is little known among young infants in America, and we may make the same remark for our own country: but the history of malignant forms of erysipelas, which have prevailed among adults in this country, will prevent us being at a loss to account for its generation in the wards of the French Foundling Hospitals. We were not aware, until informed by Dr. Stewart's note, that Messrs. Lawrence and Brodie recommend extensive incisions in the case of robust children; and although all who have seen such practice applied to adults in phlegmonous erysipelas, must admit its great utility, yet, rare indeed must be the cases in which it would be required, or permitted in young children. In like manner we dissent from the local application of the mercurial ointment, as recommended by Dr. Stewart.

5. *Rubeola* is a more common disease after, than prior to the first dentition; the anginose and cerebral complications are the most common at this early period, while those of the alimentary canal are of less importance. Anasarca or desquamation often follow. Speaking of *bleeding*, for the thoracic affections of measles, Dr. Stewart urges that it should be free and prompt, as the best preventive of the obstinate cough so likely to follow.* By neglect of this remedy a permanent hoarseness is often engendered, or even a foundation for phthisis laid. We copy the following judicious observations by Dr. Stewart, upon the use of *antimony* as a diaphoretic and expectorant.

"As a general rule, ipecacuanha is to be preferred to antimony, in very young children: for the debilitating effects of the latter are sometimes truly alarming, and have been even fatal. Still great benefit is derived from the cautious use of very minute doses of this medicine, in high excitement occurring in robust children. A grain of tartar emetic in a half-pint of water, of which a teaspoonful may be given as a dose, will be the best means of ascertaining the effect of this medicine on an infant, and it may be increased or diminished, as the child is capable of bearing its action."† 553.

6. *Scarlatina* prevailing, as it generally does, in childhood, is hardly ever seen at the Hospice, among sucking infants; yet, Dr. Stewart states, that in the New York epidemic of 1837, out of 579 deaths, 125 were between one and two years, and 72 under one year. Dr. S. treats the subject in a long and able paper in the Appendix. In regard to the *complications*, he considers, that different parts of the mucous surfaces are more liable to attack at different periods of life; and that, in young infants, it is the *trachea* which is most seriously affected—asphyxia being sometimes imminent, from the immense secretions of viscid mucus which clog the air-passages; and also congest the brain by preventing a healthy circulation. Thus, at this age,

---

* "When the cough continues annoying, after the inflammatory symptoms are removed, it is best relieved by the acetate of morphia with syrup of squills."

† Drs. Merriman and Marshall Hall concur in reprehending its use. See their edition of Underwood.

there are much seldomer found marks of inflammation in the alimentary canal, while great ulcerations often exist in the fauces and trachea, especially where depletion has been neglected. The *treatment*, he thinks, is generally conducted with too little regard to the stage of the disease, and the peculiarities of the case: he is a strong advocate for early and judicious *depletion* in the cases in which inflammatory or congestive symptoms are present: and thinks, that, when performed early, it is the best preventive of the development of the malignant form of the disease. In regard to the *anasarca*, he agrees with Dr. Stark of Edinburgh, who attributes the origin of this complication invariably to cold, and describes his success in treating it by bleeding, or when the patient is very young by abundant leeching, as almost perfect. We believe the antiphlogistic treatment is now adopted by almost all practitioners; and we may refer our readers to a case (combined with convulsions) in which it was conducted with great success and vigour by Dr. Hall, under circumstances apparently very unfavourable.*

7. *Gangrene of New-born Children.*—This is especially observed in young infants in whom the circulatory and respiratory functions are imperfectly executed; thus producing a congested state of the extremities, which turn livid and soon mortify. This he considers to be different to the gangrenous erysipelas of Underwood—arising, as it does, not from excess of inflammatory action, but from the disturbed capillary circulation of an imperfectly oxygenated blood. In these cases he has also found the lungs, heart, and venous system gorged. He recommends us to endeavour to diminish the congestion by leeching the axillæ, and to promote the capillary circulation by friction. As the alimentary canal is sometimes in an irritable or inflamed state, he deprecates the use of cordials.

8. *Induration of the Cellular Tissue.*—Common as this disease is in France, the author devotes several pages to its discussion; its rarity in England calls upon us to condense considerably his observations. The name has been given to the disease from the resistance the parts affected (face or limbs) present to the touch. This does not arise from any process of solidification of the cellular tissue, but simply from the presence of œdema fully distending it; just as a bladder, quite full of air or water, becomes hard and incompressible. Of 5,392 admissions in 1826, 240 were thus affected. The disease occurs especially in Winter, and from the first to the eighth day after birth, while, with some, it would seem to have commenced prior to birth. In some cases, various parts were attacked in succession, while, in others, the whole body was implicated. The skin usually retains the red colour of infants, and the epidermic exfoliation has rarely commenced. The progress of the disease observed no fixed rule, and it frequently was accompanied by effusion into some internal organ. In 77 cases icterus was present, but this the author considers accidental. Out of 90 post-mortem examinations, the liver was found congested or otherwise affected in 20. Of 77 cases, the lungs were pathologically affected in 34. Of 77 cases, the foramen ovale was found closed in 48, and the ductus arteriosus in 28. Out of

* Lancet, 1839, No. 848.

77 cases, in 50 there were appearances of gastro-enterite. He reports these observations in order to disprove the various theories which have been formed to account for the affection, as its dependance on disease of the liver, lungs, or alimentary canal, or on an open foramen ovale; all of which he considers as unconnected, save by accident, with the affection of the cellular tissue. His own explanation of the *causes*, is contained in the following extract.

"The predisposing causes are—1, the natural feebleness of the child; 2, a state of general and congenital plethora; 3, a superabundance of venous blood in the tissues; 4, a dry state of the skin before the exfoliation of the epidermis. The immediate causes are—1, an obstruction in the course of the blood, resulting from its quantity in the circulatory apparatus; 2, its engorgement in the cellular tissue, to which it furnishes too much material for secretion; 3, the action of external agents on the skin, which, without condensing the serous fluid, as has been asserted, are yet capable of suspending the cutaneous transpiration, and thus favour the accumulation of serosity in the cellular tissue." 153.

Upon this view of the origin of the disease, Dr. Billard confines his treatment to the relief of congestion, by local bleeding and frictions.

As to the *mortality*, he considers that it has been much exaggerated; and that death is often caused by some important internal disease, with which the affection may become accidentally connected. At the hospital, the deaths, independently of such lesions, were 50; while, when the disease is in its mild form, it is not to be regarded as a dangerous affection.

### 2. *Diseases of the Digestive Organs.*

1. *Muguet of the Mouth.*—Muguet, which is but an altered secretion of the buccal mucous membrane, must not be confounded with aphthæ. After a too great determination of blood to the parts, a viscid pellicular secretion replaces the ordinary lubricating fluid. It is seen, especially in very young infants: bad nutrition, debility, and the vitiated air of hospitals, seem predisposing causes. General symptoms are not very manifest, but phlegmonous affections, especially of the alimentary canal, are often present. Guersent considers a weak solution of chloride of soda, in a mucilaginous decoction, as the best application.

2. *Aphthæ* are produced by an inflammation of the muciparous follicles, which, invisible in the natural state, now become enlarged into small miliary tumors, which afterwards ulcerate, producing the characteristic white secretion. Sometimes the surface of the ulcers become covered with pellicles of exhaled blood, which, from the dark appearance it produces, causes them to be mistaken for gangrene. The predominance of the lymphatic temperament, is especially favourable to the production of this disease, which usually manifests itself about the period of dentition. The general symptoms, as usual with the diseases of young infants, are slight; but the child wastes away, and when the stomach and intestinal canal become affected—a common and often fatal complication—vomiting and purging occur. Ulceration of the buccal cavity may also occur independently of this affection of the follicles.

3. *Gangrene of the Mouth.**—M. Billard thus explains the production of this frightful affection: the cellular structure in young infants (probably from the as yet imperfectly regulated circulatory apparatus, and from the superabundance of serum in the infant's blood) is very prone to become œdematous. This disposes them to the formation of indolent tumefactions, which always precede the production of the eschar; the compression hence resulting and not inflammatory action, causing the gangrene; and thus we find, in the tumefied and infiltrated walls of the mouth, that gangrene first occurs where most pressure exists; viz. opposite the horizontal part of the jaw, and the dental arch. The frequent disposition, in young infants, to gangrene of the vulva and of the extremities is also coincident with a similar disposition to œdema. Again, when gangrene occurs in older children, it will be found to do so especially after the cutaneous phlegmasiæ, which are so frequently followed by general infiltration of the cellular tissue. In the *treatment* of the affection the author recommends frictions during the stage of œdema; and the cauterising the eschar, when formed, with the butter of antimony or the actual cautery. Dr. Stewart says:—

"The application, which has succeeded beyond all comparison, is the *sulphate of copper*, and the rapidity of the cure under its use is in some cases surprising. It is far preferable to the use of the actual cautery: it can be applied with the utmost facility to every portion of the diseased surface; care and attention are requisite to see that every part of the diseased part is brought under the influence of the application, as a failure might otherwise ensue. Simple ulcerations and small gangrenes quickly yield to the use of this means, and an amendment is evident from the first application. The solution should be made strong."† 570.

4. *Diseases arising from Dentition.*—Although some English practitioners may exaggerate the magnitude of the influence of this process upon the system of the child, and the benefit to be derived from lancing the gums, yet we think that Dr. Billard goes into the other extreme, by his deficient appreciation of the morbid effects of difficult dentition, and the relief to be obtained from the operation. Unquestionably, many symptoms and diseases are wrongfully attributed to the obstructions which may prevent easy dentition; but, surely, it is conformable to all analogy to suppose, that the existence of these in the susceptible frame of a young child, will frequently produce many morbid affections, and almost always complicate those already existing. His objections to rubbing the gums with hard substances, and to lancing them, are at best very fanciful; the first, in the early stages of dentition, evidently affords the child ease, and, from the increased flow of saliva it causes, benefit. Few will agree with him in reprehending it on the score of the existence of inflammatory action; for, although, at this period of life, it is true, that the gums and all neighbouring parts are freely supplied with blood, yet, is not the gum itself in a state of inflammation, nor is the process of teething in itself, at all analogous to an inflammatory process. As to the operation of *lancing* the gums, his objection to it, founded on the erroneous idea of pro-

* See Marshall Hall in Edinburgh Journal, Vol 15, and Lancet, No. 849.

† The following is the formula of Dr. Coates, whose paper in the N. Amer. Med. Journ. for 1826, is quoted by Dr. Stewart:—℞. Sulph. Cupri ʒij. Pulv. Cinchon. ʒiv. Aquæ ℥iv. bis die applicand.

ducing a hardened cicatrix, will have little weight with the practitioners of this country; few of whom pass many days without seeing ease, sleep, and improvement follow its performance too speedily and too frequently to be the work of chance. Another objection he makes, is, that disappointment frequently occurs, in consequence of the tooth not appearing shortly after the gum is lanced. We notice this, because we know that many practitioners decline lancing the gums when the teeth are not near the surface. But it is presisely in those cases, in which you have to cut down a great depth through the indurated gum and thickened capsule, before the lancet grates upon the tooth, that the most benefit is derived: the cases in which the tooth is seen almost projecting through the gum, seldom require any or but little interference. When we find the *gums spreading*, which may occur months prior to the cutting of the teeth, provided the child be suffering from symptoms of irritation, we may always be certain that they are in a state to permit and very often urgently to demand the operation. Prior to this, as long as the existence of the sharp ridge assures us that the process of propelling the teeth to the surface has not commenced, our interference would be improper. While writing this article we were called to see a child about three or four months old, whose only symptom was violent, heartrending screaming, unaccompanied with the disorder of any one function. On examining its mouth we found it had not slobbered; there was neither redness nor tenderness of the gums; but these were well *spread out*. Free incisions down to the teeth were made, and the child soon fell asleep, awaking lively and well, and so continuing for some days; when, a recurrence of the same symptoms were relieved by like means. To prevent disappointment, we should always explain to the parents our object, telling them, that, although the tooth may not appear, yet, the incision, by removing the tension and pressure, will often be productive of the greatest benefit. Dr. Billard does not consider separately the sympathetic affections resulting from dentition, referring to the parts of his work where these are described as idiopathic diseases. This, as a matter of arrangement, is right enough, yet, in treating these affections we must never overlook the state of the dentition; by paying attention to it, we may often at once relieve the most alarming secondary symptoms; while, if this point be neglected or inefficiently attended to, all the means usually effectually adopted for the relief of the disease when idiopathic, however skilfully employed, will often now be partially or entirely useless, or needlessly employed even if successful.

Dr. Billard relates two cases of *effusion of blood* into the substance of the gums, in children of two or three weeks old, which he considers to have been of a scorbutic nature.

5. *Congestion of the Organs of Deglutition.*—Of 200 children, between the ages of one and ten days, this existed in 190. The author alludes to it principally to caution against the mistaking it for inflammation, in very young children, and, as an interesting exposition of the nature of anginose affections in cutaneous diseases; for, although, in these cases, he found no relation between the congestion, and an inflamed state of the alimentary canal; yet he found a most intimate connexion between its degree and duration and that of the redness of the skin of young infants. It is to be distinguished from *inflammation* of the same parts, (of which he gives cases) by its disappearance about the 10th day, its general diffusion, and the absence of any serious symptoms.

6. *Œsophagitis.*—A rare disease among adults, is much more frequent in infants, in whom it sometimes exists as an intra-uterine affection. He thinks it is often caused by the effect of too hot food on the congested œsophagus.

A very characteristic symptom is *vomiting* immediately after deglutition, before digestion has produced any change on the milk, and great emaciation attends the disease. Dr. Billard dwells upon the fact, that writers on the diseases of children have neglected to observe that inflammation and congestion of the œsophagus may be a cause of vomiting, independently of any affection of the stomach;—a fact, he believes, furnishing an interesting elucidation of the experiments of Magendie and Maingault on vomiting.

7. *Gastric Indigestion.*—This is chiefly denoted by vomiting the coagulated, undigested milk;—not vomiting from mere repletion, but from deficiency of digestive power. Frequently, there is much acidity present, although, it is very doubtful, how far the coagulation may result from the action of the acid on the too abundant caseum. In *treating* these cases, when but slight, we should do nothing; but, when they are severe, so as to produce emaciation, the author follows the directions of Rosen; viz. the limiting the child to very small portions of food at a time, and the exciting vomiting with an oiled feather, if any symptoms of repletion exist. If, from the too great abundance of caseum, the child cannot digest the milk, artificial feeding, by means of milk and water, whey, thin pap, and gelatinous substances must be tried. If any acidity be present, a few grains of magnesia may be suspended in sweetened water; but, ever alive to the possibility of the existence of inflammation, our author is excessively cautious in recommending even such an aperient as this. Nevertheless, we believe if he employed some one rather more freely, he would find many cases of infantile vomiting disappear under its use.

8. *Gastritis.*—Great *congestion*, even to the effusion of blood, and *inflammatory* action, to the disorganization of tissue, may occur in the infant's stomach, prior to birth. Indeed, this organ (like most others) is almost always congested at birth. In regard to the symptoms of this state, the author, after saying that the digestive organs become often deranged, thus proceeds:—

"But it is not simply in the digestive functions that we must look for the symptoms from which we are to infer the existence of congestion of the stomach; let us note that this is but secondary, that it is the effect of a cause which has existed before; and it is towards the seat of this cause that our attention is to be directed. Now, every time we see a child born with all the signs of sanguineous plethora, where we observe the respiration becoming established with difficulty, and the characters which we shall hereafter indicate as being those of pulmonary congestion, all united with sanguinolent vomiting; we shall then have reason to believe the existence of a passive congestion of the stomach; because experience has proved that this lesion is, in infants, as in adults, the result of a disturbance of the circulatory apparatus. The treatment, in such cases, ought to be based upon the necessity of restoring the blood to its habitual channels, of relieving the tissue of the organs from the superabundance of this fluid; and above all, to direct our remedies to the congestion of the heart and lungs. Sanguineous evacuations are the proper means for accelerating the subcutaneous capillary circulation." 249.

Gastritis, properly so called, varies according to its seat and effects.

*(a.)* The simplest state is *erythematic*, which, however, is to be distinguished from mere congestion, both by the different distribution of the blood, and by the accompanying tumefaction and friability of the mucous membrane.

*(b.)* Gastritis with altered secretion, or *muguet* of the stomach, is much rarer than the same affection of the mouth and œsophagus. We should suspect its existence when the symptoms of gastritis supervene upon muguet of the mouth.

*(c.)* *Follicular or Aphthous Gastritis.*—The follicles "sometimes appear under the form of small, white, round, slightly projecting granulations, terminating in a black point, which marks their excretory orifices; sometimes they inflame, tumefy considerably, and finally ulcerating become disorganized. In the former case they give rise to but few symptoms; in the latter, they are accompanied by the symptoms of intense gastritis."

*(d.)* Gastritis with *disorganization;* this may be manifested, either by ulceration, gangrene, (which is very rare in infants) or gelatinous softening.

*Gelatinous Softening.*—Of this disease, first observed by Cruveilhier, Dr. Billard says:—

"In the three instances reported in this work, and in four others in my possession, I have always remarked the afflux of serous fluids towards the stomach: we have reason to conclude it always preceded the softening, and that it essentially concurred in giving the jelly-like appearance presented by the macerated and softened mucous membrane, this serum being mixed with the blood drawn thither by the inflammatory stimulus. If our conjecture be well-founded, the softening ought always to occur towards the most dependent part of the organ, for it is in that part the fluids generally accumulate; and experience proves that it is at the great tuberosity of the stomach that this disorganization occurs. This is what I have observed in the history of the seven cases of gelatinous softening in my possession, and M. Baron informs me his experience has always confirmed this remark." 266.

The parts of the mucous membrane affected are more or less circumscribed; and sometimes death is sudden from perforation. Dr. B. has met with the disease in very young children, prior to the period of the first dentition, mentioned by Cruveilhier. Following the ordinary symptoms of gastritis, there are great prostration, wasting, and restlessness.

The author directs attention to the fact, that gastritis is often *latent*, or masked by other diseases; presenting, thus, in its progress, no marked symptoms prior to a fatal termination; but he recommends that our suspicions should always be aroused, when we perceive great emaciation, accompanied by disordered digestion.

*Treatment of Gastritis.*—Dr. Billard recommends abstinence from the breast, and the support of the child by injections of gruel and milk. Without agreeing with him as to the propriety of this last practice, we may notice here, a point in his treatment of children's complaints, which practitioners in this country would do well to attend to. It is, that he insists, in all diseases of an inflammatory tendency, the child should be made abstain, more or less, from its usual supply of milk, or that this should be diluted. In this country, this point is very much overlooked, and too often, the infant is permitted, or indeed encouraged, to suck as much as it can.

The author has little confidence in the good to be derived from leeches, and only orders two or three when the symptoms are very urgent. He cautions against the use of tonics or stimuli, in the state of prostration, as it often depends upon an inflammatory disorganization.

9. *State and Functions of Intestinal Canal prior to Birth.*—Dr. Billard supposes the liquor amnii to contribute to the nourishment of the fœtus, by means of a true intra-uterine digestive process, whereof the *meconium* is the fæcal result. The digestive apparatus of a new-born infant presents the same appearance as that of an adult, who has died during digestion: the duodenum and jejunum are of a rosy hue, the villi developed, and sometimes the follicles prominent. Shortly after birth, the mucous membrane loses its florid colour, and becomes of a milky flocculent appearance, secreting an abundant mucosity. The internal surface of the intestinal canal of the new-born child, is lined with a tough layer of mucus, forming, as it were, a species of plastering. It detaches itself, shortly after birth, by a species of exfoliation, forming the little flocculi so often seen in the stools of infants, and sometimes coloured by the bile. He thinks this layer is the *saburra* of new-born children, of some authors.

10. *Imperforate Anus.*—The author relates two cases, and Dr. Stewart one. In the first, the imperforation was seated at the depth of an inch: an incision was made, which seemed to remove the obstacle, but no meconium followed, and the child died. On examination, it was found that the incision had penetrated the cul-de-sac, but a clot of recently effused blood obstructed the orifice thus made. In the second case, complete relief immediately followed; but the child died of gastro-enteritis following the operation. In Dr. Stewart's case, the orifice of the anus existed, and the occlusion was situated three-quarters of an inch above it. The operation was performed with a trocar, the meconium followed, and the child did very well. The greatest danger lies in cases of this kind; for, owing to the existence of the anal orifice, the imperforation is often long unsuspected.

11. *Intestinal Congestion and Hæmorrhage at Birth.*—" I have examined with much care, twenty-five cases of passive congestion of the intestinal tube, without hæmorrhage, in children who have died a few hours or days after birth, fifteen of them exhibiting all the signs of apoplexy—the symptoms relating to the digestive apparatus were nothing; those only were observed which related to the state of congestion of the lungs and heart; and the intestinal congestion, which was an effect of that of the respiratory apparatus, was not ascertained until the opening of the body. Although this remark may appear of a negative character, still it is worthy of attention, for we ought to conclude from it, that whenever a child is born apoplectic, the digestive apparatus will also partake of the congestion of the circulatory organs, and both ought to be the objects of our attention. I have examined fifteen cases of passive hæmorrhage—most of them were remarkable for the plethoric condition of their bodies, and the general congestion of their integuments. Some on the contrary, were pale and feeble, as is common after abundant hæmorrhage. In all, the large abdominal vessels, the liver, spleen, lungs and heart were engorged: in nine, the fœtal openings were obliterated—in the rest open. In all, the meninges of the brain and spinal-marrow were injected; in all the intestinal tube contained more or less red blood." 285-6.

In treating these cases, he recommends one or two leeches to the anus, and strongly advises, when the child is asphyxiated, the letting the funis bleed.

12. *Intestinal Indigestion—White Softening.*—Many children die of diarrhœa, entirely unaccompanied by inflammation—literally perishing, from the inability of the intestinal canal to perform its digestive functions. There is emaciation, a decoloration or blanching of the integuments, as in chlorotic females; the discharges are white, mucous, and excessively fluid. After death, the appearances are, a decoloration or blanching of the mucous membrane, in the first stage; and a softening of it in the next. The disease almost always arises from defective nutrition, in consequence of the improper suckling or feeding, to which the child has been subjected. As to its *remedy*, Dr. Billard lays great stress upon the due apportioning the milk, or other aliment, to the digestive powers of the child. He quotes several analyses of the milk of different women, to show that it differed in each of these, as to the quantity of solid and nutritious matter it contained; while that of the goat differs also from the woman's, chiefly by its alkaline nature, and by containing half the quantity of caseum. He relates a case of an infant who fell ill while copiously supplied with milk of too nutritious a quality, but who throve well when supplied with a poorer aliment. This is a point perhaps we do not attend to sufficiently: all acknowledge the ill effects of milk which is too poor upon an infant; but, the possibility of its being too nutritious has been overlooked. "I have often seen, at the Hospice, goat's milk diluted with barley water, perfectly digested by those children who rejected the milk of their nurses, and who had wasted from defects of alimentation." When the disease has gone on to the extent of softening it is incurable.

13. *Colic of Infants.*—Dr. Billard considers an exaltation of sensibility, producing colic independently of inflammatory action, very rare; and hence, he is most cautious in using antispasmodics. Dr. Stewart's views are more just: he says, that, where colic originates in cold and damp feet, &c.,* an encreased action has been determined on the mucous membrane, bordering upon inflammation; but, that it frequently arises from undigested food, &c. and may be quite unconnected with inflammation, and accompanied with much flatulence. Hence, in treating it, the due regulation of the diet, is

* Dr. M. Hall particularly alludes to the frequent production of colic in infants, by coldness of the extremities; and its disappearing upon warming them with the hand.—*Underwood, 9th Ed.*

of primary importance, while an occasional emetic is required for the removal of the undigested matters. As acidity is often present, an antacid laxative may be combined with the antispasmodic. An injection of catnip or camomiles will often at once relieve a paroxysm. Fomentations and frictions of the bowels while the feet are kept in warm water, will relieve the congested state of the mucous membrane.

14. *Spasm of the Intestines, producing General Convulsions.*—"The intestines after birth sometimes become extremely irritable, and are affected with spasms, in consequence of which the digestive functions are completely disturbed; sometimes also general convulsions or spasmodic movements of the face and limbs occur. It is often impossible to understand the cause of these convulsions, which frequently cease and re-appear with greater intensity at a period more or less remote from the appearance of the first symptoms. I have seen infants seized with these nervous colics while sucking; they quitted the breast abruptly, cried suddenly and violently; the abdomen swelled immediately, and their agitation did not cease until a quantity of gas had escaped by the anus." 320.

Dr. Billard quotes a paper by Dr. Parrish of Philadelphia,* with approbation. Dr. Parrish supposes the affection to be seated in the muscular fibres of the intestines, and particularly remarks the relief attendant upon the expulsion of the gazeous fluids which distend the abdomen. In one fatal case, he found great part of the intestines irregularly contracted; the bowels being in some places no thicker than a quill, while in others, the canal seemed as if it had been tied with a ligature. This spasm of the intestines, Dr. Billard considers as originating in irritation of the cerebro-spinal apparatus; and that it should be treated by leeches applied to the mastoid process, or by bleeding from the arm or foot. Dr. Parrish recommends stimulating frictions to the spine, the administration of assafœtida and laudanum, and the lancing the gums, when near the period of dentition; (to which formidable operation, Dr. B. enters his caveat). The removal of the constipation, and the expulsion of the gas should be facilitated; and Dr. Parrish has effected this last, by the introduction of an empty syringe into the rectum.

15. *Enteritis, Congenital.*—"I have observed ten cases in infants that have died on the first or second day after birth. In three, there was an evident inflammation of the follicular plexus of the ileo-cæcal region. In others, there existed a number of white follicles, slightly projecting, in the cæcum, surrounded by a red circle; ulceration had commenced on the summits of some of these follicles. In five others, the inflammation only consisted in red patches, with tumefaction and friability of the mucous membrane. The meconium was unaltered. The children were usually pale and thin." 299.

2. *After-birth.*—Dr. Billard arranges the varieties of enteritis, as he did those of gastritis. We will not follow him in detail, but in his description of follicular inflammation, there occur some observations, respecting the *non-inflammatory development of the intestinal follicles during dentition,* that we think well worthy of notice.

"The changes in the muciparous follicles of the intestinal tube are not all of an inflammatory character. They experience, for example, at the period of den-

* North American Med. Journ. 1827.

tition, an encrease of vital energy, which augments their secretion considerably, and renders their size larger, and their number greater, but which still does not produce any redness, &c. as is observed in inflammation. Of twelve children, in whom this state of the follicles was observed, diarrhœa existed in ten. This is the *serous diarrhœa* of authors; and every symptom leads to the belief that there is a direct relation between the development of these follicles, and the augmentation of their secretion. Most of these children had arrived at the period of dentition, so that there appeared a remarkable coincidence between the period of the appearance of the teeth and that of the organic development of the follicular apparatus. A physiological explanation may be given of this coincidence. In fact the follicular apparatus seems destined to second the action of the intestines in digestion, by furnishing the surface of these organs with a fluid, which in all probability, assists in the elaboration of the aliment. Dogs, and other carnivorous animals, where the digestive power, if I may use the expression, is truly remarkable, possess this apparatus in a high degree of development. We ought not to be surprised to find the follicles, or the muciparous plexuses augment in volume and activity at the period of dentition in man, since the organs of digestion then receive a modification which renders them fit to fulfil their functions. At the same time the salivary glands acquire a much larger size, and secrete saliva in greater abundance." 305.

This explains the occurrence of diarrhœa during the period of dentition; it should be treated by a strict regimen, for, although this development of the follicles be not inflammatory, yet is it a state of excitement more or less allied to it. These follicles are, however, sometimes found very large even at birth; and in other cases, become the seats of true inflammation.

Enteritis may terminate in softening of the mucous membrane; but this, the *inflammatory softening*, converting the membrane into a soft reddish mass, is, of course, very different to the *white softening*, resulting from improper diet.

*Locality.*—Of 80 cases of enteritis, in 30 there was inflammation in both large and small intestines; in 36, of the small alone; and in 14, of the large alone, while the co-existence of gastritis was very common.

The *Symptoms* are almost always local, and it is remarkable, that the severest, nay, mortal diseases of these organs may occur in young infants, without any general symptoms. Vomiting, diarrhœa, with dark and green stools, tympanitis and tenderness, are almost always constant symptoms, which are often accompanied by a dry tongue, and erythema about the anus. Marasmus follows, while the countenance looks aged, and puts on a characteristic, and easily recognized appearance, called the " face grippée." There is especially in all these cases a wrinkling of the skin at the forehead and root of the nose.

The *Treatment* consists in applying one or two leeches to the anus, abstinence from the breast, and the use of demulcents. If the child is in much pain, and the diarrhœa very profuse, an enema of starch with five drops of laudanum may be given; but this latter means must be most cautiously employed; for, Dr. Billard has often seen infants, from eight to twelve days old, narcotized by six drops of laudanum, administered by the rectum. Cataplasms to the belly, and warm baths are very useful.*

* Dr. Christison mentions the case of a child, aged 14 months, who died narcotized by a dose of three drops of laudanum given in a chalk mixture for

16. *Cholera Infantum.*—Of this disease, which commits such terrible ravages among the infants of America, Dr. Billard professes personally to know nothing; yet, from the perusal of the works of the American physicians upon the subject, he arrives without any difficulty, at the conclusion, that the disease is merely severe gastro-enteritis, complicated with hæmorrhage; and criticises severely the stimulating treatment adopted by Dewees and others. This is neither wise nor liberal. The American physicians having in vain combated this disease upon general principles, find themselves reduced to the treatment of symptoms, and the following out of empirical practice; and, if after a due trial, they have found this succeed better in their hands, than that which would seem to be suggested by the post mortem appearances, is it not their duty to adopt it? Our limited space, and the non-existence of the disease in this country, prevent our pursuing Dr. Stewart's able paper upon the subject. Suffice it to say, that all authors agree that, in order that this scourge of the infant population be produced, there must be co-existent a high atmospheric temperature, vitiated air, and the period of dentition; Dr. S. considers the congested state of the liver as the primary cause, this, preventing a due circulation, causes also congestion in the follicles of the intestinal canal, as is seen in post-mortem examinations:—these follicles, as shewn by Billard, being in a very excitable state at the period of dentition, when alone this disease occurs. Thus he recommends local bleeding, and the inducing the due action of the liver by small doses of calomel; as all danger is found to be at an end, when the secretion from that organ becomes natural.

17. *Congestion of the Liver.*—The liver, which is large in the young infant, is frequently very much congested at birth, so that, sometimes, its convex surface is found covered by a layer of effused blood. This is especially the case, in the asphyxia of new-born children. In some of these cases, the *bile* is found of a black, green, or even bloody colour.

18. *Bilious Diarrhœa.*—The author enters into a minute analysis of post-mortem examinations, in order to ascertain whether a changed condition of the bile be an indication of the various pathological conditions of the liver, and he concludes, that, not only are the same morbid states of that organ attended with very different appearances of the bile; but also, that the bile secreted by the healthy liver will equally vary in appearance. He next investigates how far certain forms of diarrhœa are connected with changes in the biliary secretions. Here is his opinion:

"Thus it appears that in the 48 children to whom I directed my attention, affected with diarrhœa of yellow and green matters, (and dying of gastro-enteritis,) the liver and biliary secretion exhibited very different appearances; sometimes it was healthy, often injected, and again it presented certain anatomical characters which the state of our knowledge does not permit us to refer to any class of well-ascertained diseases. What inference is to be drawn from these facts? and what theory is to be established on so uncertain a foundation? Ought we, after the example of most of the English pathologists, to attribute

---

diarrhœa; and another of a child a few weeks old who was poisoned by four drops.

gratuitously to the liver the derangement of the digestive functions? Ought these yellow and green evacuations to be regarded as an alteration of the biliary secretion? I leave it to other authors to answer these questions, when ulterior researches shall throw more light on this subject; as for myself, I will renounce my opinions only when convinced of their error,—still believing that the yellow and green dejections, whatever may be the cause of their colour, are more probably a symptom of enteritis, an affection which must be removed before the diarrhœa which is only an effect can be cured." 338.

Dr. B. is therefore quite at issue with most English practitioners; as he doubts the effect of heat in disordering the functions of the liver; he disbelieves in a diarrhœa thus originating, and denies the effect of the bile in changing the colour of the intestinal secretions. We shall not follow Dr. Stewart's long exposure of the fallacy of his reasonings. Surely our various writers on the diseases of tropical climates have amply decided the following question of the author—"Why not attempt to show by anatomical researches, that the liver, under the influence of heat, is irritated, and ceases to discharge its natural functions?"

Of course the author, explaining bilious diarrhœa as always arising from a gastro-enteritis, is horror-struck at the idea of relieving it by small and repeated doses of calomel, as recommended by Dewees and Stewart. Yet is the principle of the practice recommended sound;—preceding this medicine by a mild aperient; always being on the alert for the possibility of inflammatory complications arising; and postponing the use of astringents, until the natural colour of the discharges is restored.

19. *Jaundice of Infants.*—The following are the conclusions Dr. B. arrives at:

"1. Jaundice, being sometimes local, cannot arise from a general cause, which would extend its morbid influence over every part of the body, as diseases of the liver, for example, to which it has been usual to attribute it: and Lobstein has remarked also that, the medulla spinalis is sometimes coloured yellow, at a period anterior to the secretion of bile. 2. The liver and bile are found in cases of jaundice to be in very various conditions, and it would be difficult to explain what would be the pathological state of this organ, or of its secretion, to produce the disease in question.* 3. Notwithstanding the sanguineous congestion of the liver and integuments co-existing with jaundice in the greatest number of instances, it is probable that the retention of this fluid in the organs, and the deposition of serum, which is almost always yellow, is the cause of jaundice." 509.

He has observed this yellow colour in the brain, spinal marrow, and tissue of the lungs, when serous; also the heart and pericardium of a deep saffron

---

* We see neither difficulty or improbability in the explanation by Underwood, of cases of jaundice being caused by viscid obstructions of the biliary ducts, which yet may not, after death, always be manifest. With Dr. Billard, it seems a *sine qua non* that to refer the cause of any symptom to a certain state of an organ, you must be able, after death, to point out the lesion of that organ; which is often, of course, impossible. Seeing the ease with which jaundice in infants may be removed by a mild mercurial, we think his own explanation of it, by supposing it to arise from the colour of the serosity effused into the texture of the parts affected, is very untenable.

colour; while, shining, yellow striæ are sometimes spread through the substance of the kidney. He has found the muscles yellow, when the whole surrounding adipose structure has been white; and, on the contrary, yellowness of the adipose structure, while the skin, &c. has been colourless. The osseous system may be alone affected. He has observed the affection of the conjunctiva less often than in adults.

We must not confound with jaundice, the yellowness of skin of new-born infants, which seems a natural transition from the tegumentary congestion to the natural colour of the skin, as mentioned in another part of the present article.

20. *Peritonitis in Infants.*—This, Dr. Billard states, is much more common than is generally believed. It may not only be developed after birth, or about that period, but is often *congenital,* as manifested by the adhesions, &c. formed.* It has not been observed that children born of mothers affected with peritonitis, acquired it from them. When it occurs *after birth,*

"The symptoms peculiar to peritonitis are, tension of the abdomen, which rises in a point towards the umbilicus, restlessness, pain indicated by a pinched face, and the increasing cries of the child, vomiting, eructation, constipation, and lastly, a general sinking and smallness of pulse." 356.

21. *Ascites in Infants.*—The author says it is not uncommon to find, in the abdomen of infants who have perished slowly from chronic phlegmasiæ, a few ounces of serum, without any accompanying peritonitis. In these cases, the children are pale and feeble, with œdematous extremities: and, after death, the digestive tube is usually colourless and softened. He refers to two cases of congenital dropsy, one in Roux's Journ. de Medicine, tom. 17, in which the dropsy of the child was accompanied by the retention of a large quantity of urine by the mother, necessitating puncture of the bladder during labour:—the other, reported by Dr. Ollivier, in tom. 8 of Arch. Gen. de Med. in which the fluid was contained in the cavity of the gastro-colic epiploon.

22. *Hernia.*—Upon this subject there is no new information presented; but an interesting case is related of *congenital inguinal hernia, formed by the ovarium.* The case was involved in great obscurity until the death of the child from pneumonia, when 26 days old.

"The hernial tumor was formed by the left ovarium, which had descended through the inguinal canal and ring, which was much larger than is usual to find it in girls. The uterus, drawn by the round ligament, and by the ovarium which formed the hernia, had deviated from its natural position, and was inclined towards the left side of the bladder. The left kidney, instead of being found on a level with the other, was drawn downwards by the cellular tissue, with which it was enveloped, and also by a fold of the peritoneum, which was

---

* See some very interesting papers by Dr. Young Simpson, in vols. 50 and 52 of the Edinburgh Journal. In these, he relates many cases of intra-uterine peritonitis, and enters very fully into the causes and effects, both upon the life of the fœtus, and in the production of congenital herniæ and malformations.

intimately connected with the orifice of the sac: the renal artery had also yielded to this traction, and was elongated and narrowed; lastly, the ovarium and the fimbriated extremity of the fallopian tube, a little reddened and tumefied, were lodged at the bottom of the sac formed by a prologation of the peritoneum, with which cavity it communicated. There were no convolutions of the intestines adhering to the surrounding parts, and the ovarium of the opposite side was in its usual situation. Upon carefully examining the round ligament of the uterus on the side on which the hernia existed, I found it much shorter than that of the opposite side, and that it terminated in the labium by an aponeurotic expansion, in place of losing itself in loose filaments, as is usually observed to be the case: from which it would seem, that the ligament, shorter and more firmly fixed to the labium, had, in the first place, caused the deviation of the uterus, and afterwards drew with it the ovarim through the ring." 362.

4. *Urinary and Generative Organs.*

1. *Malformations of Urinary Apparatus.*—These are more numerous than those of any other organ; and, owing to the close connexion of the various parts of the apparatus with each other, malformations usually occupy more than one portion of the apparatus. Thus, Dr. Billard mentions a case of obliteration of the ureter, accompanied by, and causing, a true congenital dropsy of the kidney; and another, of obliteration of the urethra, followed by enormous distention of the bladder, ureters, and kidneys.

As to the *excretion of urine* in the fœtal state, speaking of these two cases, he says:—

"They will serve to prove that the excretions of the fœtus, at least those of the urinary organs, are, in the normal condition, rejected from the body, and probably are mixed with the waters of the amnios; since it appears that, when any obstacle occurs to the passage of this fluid, it reflows into the reservoirs, and distends them excessively, in the same manner as is observed in adults who are affected with a stricture of the urethra, or paralysis of the bladder." 347.

2. *Inflammation of the Bladder.*—Although Dr. Billard is not conversant with any symptoms of this affection in the young child, yet he has often found, in post-mortem examinations, well-marked signs of its existence, and to it he is inclined to refer some of those cases of retention which are usually attributed to paralysis of the bladder.

3. *Retention of Urine.*—Upon this subject, Dr. Stewart observes, that, where the secretion is suspended, and the bladder empty, we may generally re-excite it by an infusion of parsley root, and a little spt. æth. nit.; especially if we aid these by the bath and enemata. Fomentations and frictions will often relieve, when it arises from spasm of the neck of the bladder; but if these means be not successful, we must soon employ the catheter. He agrees with Dewees, that infants have been often lost from this affection having been overlooked; and recommends us always to make an examination of the abdomen, where there is the least reason for suspicion.

4. *Bloody Discharge from the Vulva.*—"It is not unusual to observe red and fluid blood flow continually from the vulva for some days or weeks after birth.

From the observations of Dr. Ollivier of Angers, this discharge, which is somewhat like the catamenia of adult females, continues sometimes a week, fifteen days, or more, without any inconvenience being experienced by the child. It is unaccompanied by redness, swelling, or any symptom of irritation. The child has no difficulty in urinating, the alvine evacuations are neither more nor less frequent than in the normal state, and the general health of the child undergoes no derangement. It would seem as if Nature had anticipated, in some sort, the establishment of the function which is developed and regulated at a later period of life." 497.

Dr. Billard says, the vagina is naturally much lubricated with tenacious mucosity; but he does not mention the case of this becoming profuse, forming, what has been called *infantile leucorrhœa.* Arising usually in a state of depraved health, this becomes, sometimes, both profuse and troublesome; and, when accompanied with irritation, it has led to very injurious suspicions.*

5. *Gangrene of the Vulva.*—This has much analogy to the gangrene of the mouth, already treated of; and is especially likely to follow the cutaneous phlegmasiæ, as erysipelas. It is sometimes very obscure, and should be early treated with cauterization. It differs from the affection of the mouth by the absence of the typhoid symptoms.

"In gangrene of the mouth, all the gaseous products of the local disorganization are exhaled at the orifice of the respiratory passages, and perhaps the pulmonary absorption of the putrid emanations of the gangrenous part may be the cause of the symptoms of debility accompanying the disease. Indeed, all the phenomena appear which arise from injecting putrid matter into the veins. In gangrene of the vulva, nothing of the kind is to be seen; for there are present a constant febrile action, cries, and restlessness, the intensity of which increases with the progress of the disorder; the disorganized part being isolated, and not in the vicinity of the opening of the respiratory organs, the inspired air is not charged with deleterious particles." 499.

5. *Organs of Respiration.*

1. *Coryza.*—This affection, though often very trivial, is, at other times, of great importance. The unimpeded state of the nares, is of infinitely more consequence to the infant than to the adult; seeing that much more of the respiratory function is performed by their agency, especially during the time of suckling. When coryza becomes very severe sucking is almost entirely prevented, and the child becomes exhausted with fatigue, pain, and inanition. The disease may be very rapidly fatal. Sometimes it is accompanied by pellicular concretions, in which cases the children may quickly sink; while, in the chronic cases, the membrane may become disorganised. Hydrocephalus sometimes follows; or gastro-enteritis may accompany it. In the *treatment* of these cases the child should not be permitted to suck, but should be

* See Percival's Medical Ethics. Medico-Chirurgical Transactions, vol, 7, p. 94. Dewees' Diseases of Children. Sir A. Cooper's Surgical Lectures. Lawrence, in Lancet, No. 363; and Locock in Cycl. Pract. Medicine, Art. Leucorrhœa.

fed, while, if the powers of deglutition be very defective, recourse should be had to nutritive injections. Emollient vapours, by temporarily encreasing the tumefaction of the parts, only add to the distress. Laxatives are required, while a blister at the nape, or on the arm is useful.

2. *Congestion and Inflammation of the Larynx.*—At birth, this organ is very much injected. Sometimes there are ecchymoses in the cellular tissue, as if from external violence; while, in others, blood is exhaled from the mucous membrane, especially in plethoric infants, and when the cellular tissue is hard and œdematous. *Laryngitis* is not so common in infants at the breast as it is in older children. The disease seldom existing alone is often very obscure; but may sometimes be distinguished by the cry, which is so faint as hardly to be heard, while the reprise is acute and predominant. Although there is not vomiting, as in œsophagitis, yet, is the millk sometimes suddenly rejected by a spasmodic cough. It is to be recollected, that when the larynx is narrow, the least tumefaction may be attended with violent spasm of the whole of the respiratory organs: a state which Dr. Billard has also found, after death, to have arisen from the collection of a large quantity of thick mucosity. He recommends in treating this disease, the use of leeches and fomentations to the throat, and rubefacients to the feet.

3. *Croup.*—Dr. Billard considers the analogy between this affection, and common catarrh proved: the membrane resulting differing only in degree of consistence from the ordinary catarrhal secretion. "It is a catarrhal phlegmasia, but the blood destined to the secretion of mucosity is, in the case under consideration, concentrated in greater abundance, or rendered plastic by inflammation, and imparts to the mucosity that portion of its composition which concretes the quickest, that is the fibrin; whence arise the striæ, pellicles, and white patches with which the mucous membranes affected with muguet or croup are covered." Young infants are not so susceptible to the disease as are older children, but are more so to other affections of the mouth and nasal fossæ; and the readiness with which symptoms of suffocation arise in young children, suffering from affections of the air-passages, render these to them almost as dangerous as croup.

*Treatment.*—The author, after detailing his mode of treatment, which contains no new points, relates, with very natural compunctions of conscience, a case in which he was induced to follow Bretonneu's proposal, of opening the trachea; to give the gasping child the remote chance of breathing through a portion of the air-tube yet unaffected? Oh, no!—that were too simple a matter: but, "to introduce a quantity of alum or calomel, to destroy and remove the mucous pellicle!" The young girl in question died during the operation.

Dr. Stewart speaks of the "uniform success," following the adoption of the plan of treatment of Dr. D. Hosack, and it seems to us a very judicious one; we present an abstract of it:—Dr. Hosack considers the differences of opinion, as to treating this disease, to have in a great measure arisen from our having neglected to observe sufficiently accurately its division into various *stages.* He himself distinguishes the three following. *First.* Wherein the affection is strictly *local;* no reaction of the general system present—the cough and wheezing inspiration, with the cries after coughing, alone denoting the impending mischief. In this stage—the *forming* one, we should, by the most active means, endeavour to restore the suppressed secretions of the trachea and pulmonary membrane, and by open bowels and open skin, to guard against the general excitement of the system. For this purpose, he employs an emetic of ant. tart. and p. ipec. repeating it until a copious secretion from the trachea and lungs result. *Second Stage.* The whole system partakes now of the irritation; fever, increase of cough, and wheezing without interval; the dyspnœa, owing to the trachea, bronchi, and lungs being overcharged with fluids is most urgent; now also, symptoms much resembling apoplexy appear. When this state of things occurs, peculiar effusions take place in the wind-pipe, bronchi, and vesicular structure of the lungs, (membranous in the two former, and a viscid fluid in the latter); after which the febrile symptoms may subside, but the local ones become more and more urgent. Here the most efficient means of diminishing the plethora of the vessels, and of diverting the irritation from the affected part, become necessary. Here then it is that free *bleeding,* repeated as occasion may require, should be had recourse to. The child should not, as recommended by Ferrier, be bled to syncope, but say we take from two to four ozs. from a child under two years, and from four to eight ozs. from a child between two and six. Partial relief immediately ensues, but we must not trust to it; and immediately after bleeding Dr. Hosack again resorts to emetics. If, after the operation of these, the symptoms still continue violent, the bleeding must be again repeated, the patient be immersed in a warm bath, a large blister applied to the throat, and calomel (gr. v. 2dis horis) pushed on until, aided by injections, it causes free purging. If still unsuccessful, calomel and James's powder may be given (gr. ij. to v. under four years) every two hours; but, when free stools have been produced, tartar emetic may be exhibited so as to excite nausea and relaxation; while, if the cough be very troublesome and the fever subsided, some laudanum may be added. *Third Stage.* From this, in which the effusions have occurred—the *membranous* stage—recovery is so rare, that it might be called the fatal stage: calomel, squills, *seneka,* ammonia, assafœtida, the vapour of vinegar and water, &c. being remedies which excite the secretions without impairing the strength, may, by persevering use, sometimes be the means of ejecting the effused matters. It is at this stage that *seneka* is useful; while it and other like remedies may be entirely improper when used, as they sometimes are indiscriminately while the organs are preternaturally excited, as in the forming and febrile stages. In this stage vitriolic emetics have been sometimes useful.

We cannot quit this important subject without strongly directing our readers' attention to a very important communication in the Dublin Journal,* by Dr. Lehman; in which he recommends attacking croup, at its very outset, with the constant and efficient external application of heat to the throat.

* This article is translated in the eighth vol. by Dr. Graves, from the Medicinische Zeitung, and consists in the recommendation of the use of hot water. In the same volume is a communication from Dr. Kirby, recommending, from long experience, the application of hot salt to the throat.

An important remedy, indeed, is this, not only for the ease with which, in the absence of the medical attendant, it may be had recourse to; but also, from the undoubted efficacy we have known attend its employment, especially when the diaphoresis it has a tendency to produce, has been accelerated by the simultaneous use of small doses of the tartar emetic. Practitioners would do well to instruct the heads of families of the important means they have at hand, in order that they might have instant recourse to it, while medical aid is sent for; and, although we may be sanguine in our anticipations of a good result in the great mass of cases; yet, surely the most sceptical will allow the remedy is at least harmless, and may serve to fill up that terrible interval which elapses prior to the arrival of the medical man :—when, to the affrighted imagination of the parent, the little sufferer's life seems ebbing away, and " nothing done."

4. *Imperfect Establishment of Respiration at Birth.*—" The experiments of Haller, and those more recently made by Beclard, have demonstrated that the child during its sojourn in the uterus, exercises in the midst of the waters of the amnios movements of inspiration and expiration; of course no air can enter during this first act of the respiratory apparatus. It is also possible for these movements to continue after birth with too little energy for the air to enter the lungs, either because the cells of the organ do not dilate, or that the bronchiæ are closed with mucus, more or less thick, adherent to their sides. *Yet the child may live in this condition for some hours, or even days;* and if the lungs be examined after death, not the *least trace of air* will be found in them. I have had the opportunity of examining six infants, who had lived without air having penetrated their lungs in sufficient quantity to prolong their lives. They were all remarkable for extreme feebleness, slowness of their movements, and the peculiar alteration of their cry, which consisted only of an acute hiccup. 399.

Such cases, which may properly be called, "*feebleness of birth,*" are quite opposed to cases of true asphyxia, and would be much injured by bleeding. Free exposure to the air and friction are the chief means to adopt for their relief.

5. *Asphyxia of new-born Infants.*—This, depending upon a congested state of the heart and vessels, is a very different condition of the system to the foregoing. The congested state of the heart and lungs with which some children are born, prevents the air having due access to the air-cells; and, as it is only encreased by the attempts at crying made by the child, these should therefore not be encouraged. The funis should be divided, and permitted to bleed freely ; and, if the congestion continue, leeches to the axillæ may be useful.

6. *Congestion of the Lungs, and Pulmonary Apoplexy.*—The young infant's lungs are often much congested; and it is not always easy to draw the line of distinction between the effects of congestion and those of inflammation. Engorgement is most frequent at the posterior parts of the lungs, and oftener at the Hospice on the right side, owing to the habit the nurses have of laying the child on that side. In distinguishing this affection from hepatization, it should be remembered, that the heart and its vessels are always much congested. The symptoms are often very obscure; we find a bloated face, smothered cry, laborious respiration, and percussion* dull. There often results from this congestion, a circumscribed effusion of blood into the tissue of the lungs, forming *pulmonary apoplexy*, a disease much more common in young infants than in adults. In *treating* the affection, the author says—"The first indication to be fulfilled is the abstraction of blood, which can best be done by one or two leeches under each axilla; the subcutaneous venous plexuses in this region communicate directly with the vessels of the thoracic cavity, and are thus convenient for the sudden evacuation of blood from the affected part. It is of the greatest importance to avoid clothing too tightly a child that presents these symptoms, either at birth, or at a more advanced age, for by impeding the dilatation of the thorax, there is danger of augmenting the congestion." 406.

7. *Pneumonia.*—This differs in its *nature* from the affection in adults.

"Pneumonia of infants exhibits peculiar characters. Instead of being an idiopathic affection, arising from irritation developed in the pulmonary tissue under the influence of atmospheric causes, which often excite this disease, the pneumonia of young infants is evidently the result of a stagnation of blood in their lungs. Under these circumstances, this blood may be regarded as a kind of foreign body, and it concurs in producing an alteration in the pulmonary tissue with which it combines, and is identified with it, so as to form what is called a hepatization of the lungs. In proof of this, it is known that pneumonia almost always follows congestion or engorgement of the lungs, and affects the right lung much the most frequently in the children of the hospital. Besides, the inflammation of the lungs is ordinarily very circumscribed, and is found almost constantly confined to a point previously engorged, and the pleura, which in the greatest number of instances is inflamed at the same time with the lungs at a more advanced age, is not affected in infants." 406.

As to the *treatment*, it consists in the application of from two to six leeches to the base of the chest, or under the axillæ;—the substitution of weak milk and water for the breast;—dry cupping, or blistering the chest or arms;—counter-irritation of the extremities;—purgatives.

8. *Bronchitis.*—Although ordinarily pointed out by symptoms evident enough, this may exist in a *latent* form.

"I have, in four instances, seen the remote ramifications of the bronchiæ very red and filled with thick mucosities in the bodies of children who had died eight or ten days after birth, and where there had been neither râle nor cough during life. In two of these children there was pneumonia with pulmonary engorgement; in two others the lungs were healthy, and death occurred from intestinal phlegmasiæ." 416.

*Chronic Bronchitis—Bronchial Catarrh.*—This is sometimes very tedious, and is often attendant on some form of phlegmasia of the pulmonary tissue,

* Dr. Billard considers *percussion* a more useful means for the detection of infantile disease than auscultation, especially when performed as by M. Baron, who has acquired great tact in, and powers of diagnosis from its employment. He suspends the child, by applying one hand to the anterior part of the chest, and strikes small blows with the index and middle finger of the other, upon its posterior and lateral parts.

or even on the formation of tubercle; it is also often co-existent with gastro-enteritis. It sometimes, however, continues nearly during the whole period of suckling, without any great detriment to the health, and need not cause alarm as long as emaciation does not occur. All the symptoms of suffocative catarrh are, however, sometimes present.

In the *treatment* of bronchitis, Dr. Billard recommends very similar means to those adopted for pneumonia, with which it is often combined. This is contrary to our experience; for we have constantly found, that infantile bronchitis, if seen early, may be removed by ipecacuanha, given first in emetic and then in nauseating doses; applying a blister, for two or three hours, if the case is obstinate, and the skin cool. Leeches, however, are of course required, when the case becomes complicated with pneumonia.

In *chronic* cases, Dr. B. recommends blistering between the shoulders, and the use of the balsam copaiba; of this last he has however no experience, but quotes a paper by Dr. La Roche,* speaking highly of its use; but we believe, Dr. La Roche's observations were confined to adults.

9. "*Pleurisy* is more common in young infants than is generally believed; it often appears without the lungs participating in the inflammation." Its diagnosis is difficult; but Billard places considerable stress upon the circumstance of the *cry* continuing entire while the percussion is dull. The affection may become chronic in even very young children.

10. *Œdema of the Lungs.*—This frequently occurs in cases of induration of the cellular tissue. Although it usually causes very laborious respiration, yet Dr. Billard has found it to a considerable extent after death, no symptom denoting it during life; in such cases, he supposes it occurs only at the period of dissolution.

11. *Hooping Cough.*—Speaking of its *nature* Dr. B. says—

"It is difficult for us positively to ascertain the nature of hooping-cough, but we may still obtain some knowledge of its principal characters. Thus it is evident that it is a bronchial catarrh. This catarrh however has something peculiar: the cough which it produces is always suffocative, convulsive and paroxysmal. This nervous complication is to be noticed, for here its specific character commences, and we can see it, but are unable to explain it; yet I will make one remark upon this nervous complication; it is, that, in adults as well as in children, affections of the larynx, trachea, and also of the bronchiæ, often give rise to a sudden local or general spasmodic irritation, characterized by spasm of the affected organ, or by general convulsions. Tonsillitis, angina, croup, foreign bodies in the trachea, tumors compressing the air-tubes, produce a cough more or less suffocating, very remarkable for its remissions, and which has, in some cases, a striking resemblance to hooping-cough. Admitting, therefore, the specific nature of the catarrh in this disease, and that it consists especially in a nervous complication, we are disposed to the opinion that, in many other instances, the diseases of the same organ may exhibit very evidently a nervous complication; whence it will follow, that if in a similar complication consists the specific nature of hooping-cough, the seat of the disease and the physiological lesion which exists between it and the nervous system, may concur

* North American Med. Journal, No. 6, p. 34.

in a manner that will produce the specific quality of the disease in question. The same disease, in different parts of the system, often presents various characters: different diseases having the same seat, sometimes exhibit analogous characters; the seat of the affection then has something that imparts a specific quality to diseases in general, and ought to be considered when treating them. There is also another circumstance which ought to be considered as peculiar to hooping-cough—the co-existence of mucous vomitings affected by the cough. It is explained by the relation existing between the mucous membrane of the bronchiæ and that of the stomach, and the frequency of the cough very naturally accounts for the frequency of vomiting." 426.

*Treatment.*—Dr. Billard recommends vigorous antiphlogistic treatment in the first stage, which he considers as purely inflammatory, and narcotics and antispasmodics in the second. He disapproves of emetics, and says that Guersent has found equal parts of zinc, belladonna, and cicuta (commencing with gr. ¼ ter) very useful. Drs. Stewart and Eberle, also agree with the Germans, as to the value of belladonna, especially the formula of Dr. Kahleiss.*

### 6. *Circulatory Apparatus.*

1. *Obliteration of the Fœtal Openings.*—As the most important facts stated under this head are now embodied in works of medical jurisprudence,† we will only cursorily notice the author's conclusions, from his numerous examinations:—

"From this exposition, it is evident that the fœtal openings are not obliterated immediately after birth; that the period at which this occurs is very variable, yet the foramen ovale and ductus arteriosus are usually closed on the eighth or tenth day. It results also that the modifications which follow the cessation of fœtal life, in the circulatory organs of a new-born child, occur in the following order: the umbilical arteries are first obliterated, then the vein, next the ductus arteriosus, and lastly the foramen. The pre-existence then of the fœtal openings for some days after birth ought not to be considered as a disease, since it is not uncommon to meet with it without its having given rise to a single symptom. This irregularity or tardiness is attributable to the mode of obliteration." 440.

He attributes the *establishment of the new circulation* to organic changes in the parts concerned. Thus, long prior to birth, the foramen ovale has gradually been undergoing change, so as to offer more and more obstacle to the passage of the blood; while, there is gradually deposited in the umbilical arteries, and ductus arteriosus, elastic matter, which, by its contractile power, impedes the farther progress of the blood; producing what the author calls an *active obliteration*, as contrasted with that of the umbilical vein and venous duct, which is a mere *passive* obliteration, resulting from absence of blood. Now, these organic changes may occur prematurely, or tardily; producing in different children different periods of obliteration.

---

* ℞. Pulv. rad. belladon. gr. iv.; pulv. Doveri, gr. x.; lac. sulph. ℈iv.; sach. alb. ʒij.; M. divid. in ch. 20, cap. i. 3tis horis. This is for a child two years old, and between each dose, a teaspoonful of the following mixture is to be given. ℞. Aq. chamomile ℥j.; syrup. ʒij.; acid Prussic Vauqul gtt. xij. M.

† Beck, 5th Edit. Ryan, 2nd Edit.

*The new-born infant does not require a perfectly oxygenated blood:*—

"The accomplishment of these phenomena of transition must doubtless be attended with an incomplete oxygenation of blood, since all this fluid which the heart propels to the different parts of the body, has not passed through the lungs. But, after all, is it necessary that the blood of an infant just born should be oxygenated equally with that which passes through the arteries of an adult? Would it not rather appear, that the tender frame of a new-born child ought not to receive blood possessing too great stimulating properties; that the materials of nutrition should not be too suddenly charged with exciting principles, the action of which, on the organs of an infant, may be injurious to its health, and to the progressive establishment of independent life? I am of this opinion and do not think its correctness can be denied, flowing as it does from the anatomical examination of the circulatory organs of the young child. This assertion is supported by another remark;—the lungs would be exposed to fatal congestions, if the pulmonary arteries should suddenly throw into them all the blood which flows into the heart. The ductus arteriosus, by permitting the blood to pass through it, comes, as it were, to the aid of the respiratory organs, the congested state of which will not permit the air to arrive freely in the cells. The establishment of independent life is, therefore, actually promoted by the continuance of fœtal life." 443.

2. *Aneurisms.*—These are very rare in the infant; but Dr. Billard mentions two cases. 1. *Passive aneurism of the heart,* in which "the heart was about the size of a hen's egg: the ventricle and auricle of the right side, formed, as it were, the volume of the organ. Their cavities were very much dilated, and their walls almost as thin as a sheet of paper; while the opposite cavities were very much contracted, and their walls hypertrophied." 2. *Aneurism of the ductus arteriosus.*—In this, "the heart was larger than is usual for an infant at birth; the two lateral cavities were nearly equally dilated, and were filled with black blood; the ductus arteriosus existed in the form of a large cherry-pit; its transverse diameter was about three lines and a half, and its circumference nine: from an external examination it appeared to open widely into the aorta; this size, however, existed only exteriorly, for the interior of the tumor was filled with fibrous clots, organized and disposed in layers, as is seen in aneurismal tumors of adults, leaving in the centre a very small hole."

3. *Colour of the Heart.*—Externally, in young children it is of a deep red, and when very pale, it is in an abnormal state. Internally, also, it is of a more or less deep red, the right cavities being much more so (viz. of a violet or logwood hue;—a colour which is not produced by putrefaction, even when advanced,) than the left. The vascular system, generally, is turgid, and ecchymoses or effusions are often produced: but when the blood is cleared out of the vessels, they then appear of a whitish colour.

4. *Pericarditis.*—"If inflammation of the proper tissue of the heart is rare or difficult to prove in young infants, inflammation of the pericardium is more common. Perhaps it is more frequent at the first period of life than at any other: in nearly 700 post-mortems at the hospital, I have seen seven well marked cases."

### 7. *Cerebro-Spinal Apparatus.*

1. *Gradual Development of the Brain in the Infant.*—"It appears, therefore, that, from birth to the age of one year, the brain of a child is in a true state of *transition*, and that this organ, scarcely perceptible in the beginning, reaches its proper organization about the 9th or 12th month. Is it not owing to this modification occurring in the brain of a child, that the frequency of cerebral affections at this age are to be ascribed? Not only has the brain undergone, during the first year, these organic modifications, but the exercise of its functions has also increased; it has gradually acquired its control over other organs, and it has become fit to receive from them the sympathetic indications with which it was before unaffected; it is the centre and regulator of the sensations, and this influence is felt even in disease; we frequently see, during the first period of life, great alterations in the organs, unaccompanied by any febrile re-action, any general symptoms, or any morbid sympathy; but at the age of which we are now speaking, every thing assumes a new aspect; fever, which is scarcely ever seen in new-born children, here makes its appearance on the slightest cause; hence the restlessness, cries, spasms, nervous mobility so common, so easily excited, and at the same time so transient, in children who have passed the period of infancy. These considerations will prove to us the difficulty of studying the diseases of early infancy; the cause is evidently in the organic imperfection of the encephalon, which cannot reveal to us the signs and external symptoms of these diseases." 456.

2. *Congenital Malformations.*—This section contains an interesting resumé of what is known on the subject, but no particular novelty. We may, however, notice two cases of anencephalia, without absence of the cranial cavity. The one, in whom the forehead and summit were much flattened, lived for forty days, during which time crying, respiration, and suckling were performed without difficulty:—"after death, we found only the cerebellum, thalami, the third and fourth ventricles. The fornix was separated at its middle. The posterior part of the hemispheres was sufficiently developed, but they were deficient anteriorly, leaving exposed the anterior part of the lateral ventricles." The other case lived three days, and "on opening the cranium, instead of finding the brain regularly formed, there was only a sac formed of the meninges, on the surface of which the vessels appeared as usual; this sac contained a bright yellow fluid, liquid and inodorous, like serosity. When it had flowed out, the cerebellum could be seen at the bottom of the cranium, covered by the tentorium, the rudiments of the falx cerebri, the medulla oblongata, the thalami, and the corpora striata; on the outside of these floated some pulpous fragments, which appeared to be the rudiments of the cerebral hemispheres. The pia mater, which formed the internal tissue of this cerebral cyst, was covered here and there with a great number of pulpous and cerebriform flakes."

3. "*Congenital Hydrocephalus* is, in all probability, the result of an inflammation of the meninges during intra-uterine life, or of some malformation difficult to ascertain; bearing some resemblance to a nutritive hypertrophy of the encephalon. This idea is favoured by the development of the cerebral mass and the bones of the cranium in hydrocephalus fœtuses. These bones acquire a breadth and thickness, not only resulting from inflammation of the meninges, (which cannot alone account for the phenomenon), but it evidently shows an increase of nutrition, which may be regarded as one of the causes of hydrocephalus. It is worthy of remark that, after birth, children, where the brain and cranium are very much developed, are also much exposed to hydrocephalus." 467.

4. *Congestion of the Infant Brain.*—" Injection of the meninges of the medulla and of the brain, is so common in infants at birth, that it has appeared to me more proper to consider it as a natural, rather than as a pathological state. It is found in most dead bodies; vascular injection, and even effusion of blood at the inferior and posterior extremity of the spine, are very frequent. I have often seen it without its having given rise, during life, to any appreciable symptom." 473.

If, however, this injection be much prolonged, exudation of blood, and consequent pressure will occur, producing a true *apoplexy*. Sometimes the hæmorrhage is very circumscribed, of which Dr. Billard has met with a case in a child, who died apoplectic the third day after birth; and he cites a case by M. Berard, who found in a fœtus of 8½ months, a clot about the size of a nut, lodged in the substance of the brain.

5. " *Non-Inflammatory Softening.*—This is a lesion peculiar to the encephalon of new-born infants, and is the result of congestion. This is a species of softening either general or local, which, far from presenting the characters of inflammation, on the contrary, presents all the signs indicating decomposition, and we might almost say putrefaction of this organ." Although it would seem often to result from congestion, yet may congestion exist without it, and it without congestion. It may be partial or general, involving all parts of the brain, except the meninges, and yet, " notwithstanding this destruction of the encephalon, children will live some days, not possessing simply, as is vulgarly thought, a mere breath of life, but actually respiring, crying and sucking: this occurs when the disorganization is arrested at the medulla oblongata, which remains unaffected, and which, with the medulla spinalis, controls the phenomena of life, even preserving it for some time." 475.

This softening is sometimes congenital. The *symptoms*, arising from these congestions, are a great prostration of the powers, with a congested state of the surface and of the respiratory organs; and he recommends bleeding from the funis, and the application of leeches afterwards to the occiput, if required.

6. *Inflammation.*—" It is without doubt a great advantage that the brain in children is one of the last of the viscera that becomes organized; for, if at the period of birth, it possessed all the organic and vital properties which is observed in the digestive or respiratory apparatus, it would be exposed to frequent inflammations. But its pulpous, and we might almost say its inorganic state, renders it but little disposed to phlegmasiæ, which might be developed after the congestions, of which it is always the seat at the time of birth. I can affirm that well-marked inflammation of the cerebral substance is rare in new-born children; that of the meninges is more common." 476.

Sometimes, as the result of inflammation, the cerebrum or spinal marrow become indurated, at others softened; the former state especially occurring when the inflammation has made little progress. Softening of the spinal cord rarely exists without softening of the brain; but the reverse of this observation does not hold: the softening is accompanied by a decided odour of sulphuretted hydrogen. The symptoms of prostration are more evident in proportion as the spinal-marrow becomes implicated, until when they are often not very obvious. "It may be laid down as a general principle, that extreme softness is always attended with a general paralysis or loss of sensibility; and when the consistence of the nervous tissue is encreased, there are either convulsions or an exaltation of sensibility." Induration and softening of the medulla may co-exist in different parts. The author has placed inflammatory and non-inflammatory softening in different sections, but he has given us no marks whereby to distinguish the one from the other. Indeed we doubt not that such distinction is often nearly or quite impossible. He observes, that however able we may be to distinguish *meningitis* by the accompanying convulsions or cries, yet, as *encephalitis* often presents no decided symptoms, we cannot, in young infants, decide upon its existence, when uncomplicated with meningitis, which however it rarely is.

7. *Convulsions.*—These, Dr. Billard considers as almost always dependent on a greater or less degree of meningitis, although this may not be manifested after death by other appearances than a slight injection. Speaking of the insufficiency of the morbid appearance in many of these cases, he says: "Besides, as it is much more common to find convulsions in infants with meningitis, than to meet with them without this inflammation, analogy might produce the conviction that the convulsions of children, whatever be their form or degree, whether known as spasms, cramps, twitchings, &c. all arise from cerebral or spinal meningitis. This opinion has been fully demonstrated by M. Brachet of Lyons."*

This same reasoning by *analogy*, which, when rigorously conducted, may be the means of great elucidation and discovery, when loosely employed may lead to grievous error. The opinion held by our author is one which was also maintained formerly by a celebrated writer of our own country, and which, after doing infinite mischief by the indiscriminate depletory measures it sanctioned, is now happily exploded. Every body acknowledges that some of the worst forms of infantile convulsive diseases have their seat in a diseased brain; but it is also known, that the great bulk of such cases originate from causes less serious and more removable.

8. *Tetanus.*—Dr. Billard has met with two cases in young infants; in both of which was found effusion into the arachnoid of the medullary canal.

### 8. *Organs of Locomotion.*

*Congenital Luxation of the Femur.*—This section contains a resumé of the opinions of Dupuytren. The affection may exist upon one side only, though usually it is present on both. It is not usually observed at birth, but only as the pelvis encreases in width; "it is then that the want of support of the upper part of the body on the pelvis, and its inclination forward, the hollowness of the back, projection of the abdomen, arched movements of the extremities, defect in the flexibility of the head of the femur, its

* Memoire sur les Causes des Convulsions chez les Enfans, 1824.

alternate elevation and depression in the external iliac fossa, &c. begin to be very evident."

As to the mode of its production:—

"This dislocation is generally upward and outward, and the head of the bone rests in the external iliac fossa. According to Dupuytren, this displacement appears to arise from the habitual position of the legs of the fœtus in the uterus. The thighs are closely flexed upon the abdomen, and the heads of the bones are continually pressing against the posterior and inferior part of the capsular ligament; this unceasing pressure, although without any effect in well-formed individuals, may produce a dislocation in others whose tissues are less resisting."* 492.

9. *Lymphatic System.*

"Diseases of the lymphatic system are not so common in the first eight or ten months as they are after that period. I will not, therefore, stop to describe them, but will simply remark that the lymphatic glands of the mesentery, which so easily become affected with chronic inflammation, and even with tuberculous disorganization, in children above the age of a year, affected with chronic inflammation of the intestines, do not in very young infants become the seat of phlegmasiæ; but the only alteration observed is a slight tumefaction; and when the gland is cut, it is found to be a little more condensed than natural, and of a rose colour. The changes which age effects in the nutrition and texture of these organs dispose them particularly to chronic inflammation, known under the name of *tabes mesenterica.* I do not say that infants at the breast are exempt from this disease, but that it is of rare occurrence. The lymphatic ganglia of the neck and those surrounding the bronchial divisions and root of the lungs are, in young infants, much more frequently inflamed or enlarged than those of the mesentery. The mesenteric ganglia are but slightly developed at this period of life; but they increase considerably in the course of a year; and it is to be observed that their diseases and alterations become more frequent in proportion as their development acquires a marked predominance in the system." 500.

10. *Ophthalmia of Infants.*

We rest at this section, which is a very meagre one, only for the following remark:—

"Opacity and softening of the cornea are not produced by purulent ophthalmia alone. I have seen several children, who had been reduced to complete marasmus by gastro-intestinal diseases of long duration, affected, without palpable inflammation, with softening of the cornea, which was followed by perforation and discharge of the humours of the eye. This species of spontaneous softening reminds me of the fact noticed by M. Majendie in a dog, which, being fed for a long time with sugar only, died after having been reduced to great emaciation. Was defect in alimentation the cause of the softening of the cornea?" 505.

11. *Bloodletting in Infants.*

Dr. Billard makes no remarks upon this subject; but, judging from the treatment of his cases, he uses it very sparingly; seldom ordering more than

* See Dupuytren Repertoire d'Anatomie, T. v. and Leçons Orales: also a valuable paper upon this subject, by M. Gerdy, translated in No. 848 of the Lancet.

two or three leeches. Dr. Stewart pursues the subject at some detail in the Appendix, deprecating the modern substitution of leeches for the lancet. He remarks, that the assimilative functions are very rapid in the infant, and that thus abundance of blood is formed, and the child very liable to congestive and inflammatory diseases; which are unattended with those complications, which in adults often forbid bleeding. It is the more called for, because, in them, the progress of inflammation is very rapid. He thinks, that the danger of the occurrence of convulsions has been greatly exaggerated; and that, when these have happened, the bleeding has been either carried too far, or was not required by the nature of the case. The most alarming cases of prostration from the loss of blood, with which he is acquainted, have arisen where leeches, not the lancet, were employed—an observation in which the experience of most practitioners will bear him out. The evils which might otherwise attend general bleeding in infants may be obviated, by its early employment, the careful proportioning of the due quantity to the necessity of the case, and, when nervous symptoms or exhaustion manifest themselves, the prompt use of a mild opiate or Dover's powder.

"Another reason has been mentioned for preferring local to general bleeding—it is the difficulty of finding a vein in the arm; this, with a little care and practice, need not exist. If, after applying the bandage, the part of the arm where the vein lies be gently pressed and kneaded in order to empty the cellular tissue lying over it, the vessel will in a short time be brought nearer the integuments, and may be distinctly felt, if not seen. A vein in the back of the hand may often be found, if there should be any difficulty in discovering one in the arm; or a vein may be opened in the foot. In very young children, a leech applied to the back of the hand after bandaging the arm, will often answer perfectly well." 576.

Dr. Stewart by no means proscribes topical bleeding, which is often the only kind that can be employed; and he especially approves of Dr. Billard's plan of leeching the axilla in pulmonary congestion.

Although we agree with him, that general bleeding is too much neglected in young infants; yet, we are fully alive to the dangers of its indiscriminate use, in affections so difficult of diagnosis, and at an age in which (as we think, contrary to Dr. S.) depletory measures are so ill born. For the prevention of mischief from this source, or for remedying it when it has occurred, we shall find Dr. Marshall Hall's observations very useful.*

### 12. *Viability of Infants.*

This Essay is a most excellent and critical examination of how far the various malformations and congenital diseases, which the new-born infant may be afflicted with, tend to interfere with its viability. It is founded upon the numerous pathological observations contained in the foregoing part of the work; and may be perused, with the greatest advantage, by the student in medical jurisprudence.†

---

* See his Essays on Bloodletting, and his edition of Underwood.

† An abridgment of the conclusions is contained in Ryan's Medical Jurisprudence, 2d edition.

Professor Francis, of New York, has furnished to the Appendix a paper upon this subject, for the most part highly laudatory and confirmatory of the views of Billard; but, as he justly remarks, Dr. B. has omitted stating at what period of gestation viability may commence. After alluding to Dr. Rodman's celebrated case, Dr. Francis makes the following observations, which are very applicable to a discussion upon the subject, noticed in a recent number of this Review.*

" I am the more inclined to confide in the accuracy of Dr. Rodman's statement from having seen a remarkable instance of a similar kind. In October last, 1838, I was requested to meet in consultation in a case of retained placenta; the patient, from fatiguing exertions, had been prematurely seized with labour-pains, and, after unavailing measures of prevention, was delivered, in the twentieth week of gestation, of a male fœtus, which by distinct respirations sustained life one hour. After death it was found to weigh, one pound, six drachms: its length was ten inches, and it was well formed. Too many circumstances conspired to render the age of the fœtus doubtful; it was the product of a first conception, and the parties were beyond suspicion.

My friend Dr. Wm. Barron favoured me several years ago with a fœtus which was spontaneously protruded between the seventh and eighth month of gestation, the weight of which was little more than five ounces. The cause of so extraordinarily immature and limited growth at this advanced period, was ascertained to be a disordered condition of the cord, which impaired the fœtal circulation. I give you this case because it is calculated to lead us, in all inquiries of this nature, where we might conclude too hastily as to the age of the fœtus by its bulk alone, to advert to the condition of the cord; inasmuch as an abnormal formation of this connecting link between the mother and child, as well as the sound or disordered development of the placenta, must exercise a controlling influence on the vascular energies, and more or less circumscribe the growth and dimensions of the fœtus. There is much that might be said on this subject, and in Cruveilhier we find a number of observations illustrative of the various causes which disturb that functional reciprocity which in the pregnant state is indispensable to the sound and full formation of the uterine product. It is worthy of remark, that in the case published by Dr. Rodman no notice is taken of any peculiarity in the cord, and in that which I have given its healthy structure was obvious.

I will add another case illustrative of the early period at which extra-uterine life may occur, and in which the viability has secured the subject now some seven years. Mrs. B. had been delivered, after protracted sufferings, of a dead male child, at the ordinary term of a first pregnancy. Twenty months after, the inconveniences of a second pregnancy were so great that she was on several occasions threatened with abortion. Neglecting the precautions recommended her, she had, during the prevalence of the Asiatic cholera in 1832, indulged in eating freely of Indian corn, which created much annoyance in the stomach and bowels, and in the opinion of several of her friends it was thought that this indulgence was the exciting cause of her premature labour. By one powerful effort, the entire ovum was expelled. Arriving at this crisis, I had the whole immersed in a vessel of tepid water, and having rendered the mother more secure and comfortable by a bandage, forthwith ruptured the membranes, when to my surprise I perceived a fœtus, apparently of some five months and upwards of growth. The cord was divided, and more than usual care taken with the child; a fillet or ribbon was applied round its head, which seemed unusually

* Medico-Chirurgical Review, for October 1839, p. 424.

large, and the body wrapped in cotton. By unremitting attention on the part of a competent nurse, the fondest wishes of the parents were ultimately realized, and the daughter, in the enjoyment of excellent health, has, at the present writing, completed her seventh year. Subsequent enquiry with the parents concerned made the age of this premature offspring at birth, a fœtus of the twenty-third week of pregnancy." 612.

In concluding our review of Dr. Billard's work, we again bear our testimony to the variety and importance of the facts it contains; and to the general soundness of the deductions he has carefully drawn from them. But, besides the various points upon which, in the course of this article, we have found it necessary to differ from the author; we must observe that, those who may expect to find in his book a complete dissertation upon the diseases of children, will be disappointed. For, not only does his injudicious limitation of his observations to sucking infants, exclude the consideration of several diseases of early life; but, many of the diseases to which even sucking children are liable, are either entirely omitted, or very imperfectly treated of. Upon the subject of treatment the book is also very defective. It should have rather been termed a treatise on the pathology of very young infants, and on points of medical jurisprudence connected with them. Thus, although in the discussion of questions as to the exact state of the various organs in infantile diseases, Dr. Billard's work must always be referred to as a great authority; yet, our own authors, more empirical and less scientific though they be, are more useful guides in the practical treatment of these important affections. Again, we will venture to say, that the author has sometimes attached too great importance to the appearances after death; we are convinced that in the young infant they are not always of the same value as in the adult, being much more easily confounded with the congestive state, which seems almost the natural condition of many organs in early infancy. Hence we find on his part, a degree of timidity in the administration of aperients and tonics, by no means justified by actual experience.

Of the manner in which Dr. Stewart has executed his task, we can speak in the highest terms. He has not only rendered the author's meaning faithfully, unaccompanied by the Gallicisms, which it would generally seem to be the aim of our translators to introduce rather than to avoid; but, he has, in some measure compensated for the deficiencies of the author by a very able Appendix upon the treatment of various diseases, and by the addition of a large collection of formulæ.

